# The dynamic relationship between cognitive function and walking speed: the English Longitudinal Study of Ageing

**DOI:** 10.1007/s11357-014-9682-8

**Published:** 2014-07-05

**Authors:** Catharine R Gale, Michael Allerhand, Avan Aihie Sayer, Cyrus Cooper, Ian J Deary

**Affiliations:** 1 Centre for Cognitive Ageing and Cognitive Epidemiology, Department of Psychology, University of Edinburgh, Edinburgh, UK; 2 MRC Lifecourse Epidemiology Unit, University of Southampton, Southampton, UK; 3 MRC Lifecourse Epidemiology Unit, Southampton General Hospital, Southampton, SO16 6YD UK

**Keywords:** Cohort studies, Cognitive function, Walking speed, Ageing

## Abstract

Cross-sectional studies show that older people with better cognition tend to walk faster. Whether this association reflects an influence of fluid cognition upon walking speed, vice versa, a bidirectional relationship or the effect of common causes is unclear. We used linear mixed effects models to examine the dynamic relationship between usual walking speed and fluid cognition, as measured by executive function, verbal memory and processing speed, in 2,654 men and women aged 60 to over 90 years from the English Longitudinal Study of Ageing. There was a bidirectional relationship between walking speed and fluid cognition. After adjusting for age and sex, better performance on executive function, memory and processing speed was associated with less yearly decline in walking speed over the 6-year follow-up period; faster walking speed was associated with less yearly decline in each cognitive domain; and less yearly decline in each cognitive domain was associated with less yearly decline in walking speed. Effect sizes were small. After further adjustment for other covariates, effect sizes were attenuated but most remained statistically significant. We found some evidence that walking speed and the fluid cognitive domains of executive function and processing speed may change in parallel with increasing age. Investigation of the association between walking speed and cognition earlier in life is needed to better understand the origins of this relation and inform the development and timing of interventions.

## Introduction

Maintaining cognitive and motor capabilities with increasing age is crucial for living independently, carrying out everyday activities and quality of life. There is considerable inter-individual variation in how these capabilities change with age. While some domains of cognitive function, such as verbal ability and general knowledge, show little change with advancing age, in general, fluid cognitive abilities, such as reasoning, executive function, processing speed and memory decline from midlife or even earlier (Park and Reuter-Lorenz 2009; Singh-Manoux et al. 2012). Gait is a complex motor function that tends to deteriorate in later life, although it is unclear how early in the life course these changes start. Usual walking speed is the aspect of gait that is most commonly assessed. Most longitudinal data on walking speed are based on people aged 60 or more, but comparisons of different age groups suggest that, like cognition, walking speed too may start declining before midlife (Bohannon 1997; Cooper et al. 2011; Himann et al. 1988). Poorer performance on tests of fluid cognitive abilities, slower usual walking speed or faster decline in either cognitive or motor capability are all associated with increased mortality in older people (Backman and MacDonald 2006; Studenski et al. 2011; White et al. 2013).

Evidence from cross-sectional analyses shows that there is a low to moderate correlation between scores on tests of fluid cognitive ability and walking speed, such that older people with better cognition tend to walk faster (Holtzer et al. 2006; Killane et al. 2013; Martin et al. 2013; Rosano et al. 2005; Soumare et al. 2009; Watson et al. 2010). These findings seem to apply particularly to performance on tests of executive function, processing speed or attention—thought to be of particular importance for planning and execution of movements (Yogev-Seligmann et al. 2008). Establishing whether these associations reflect an influence of fluid cognition upon walking speed, vice versa, or a bidirectional relationship and whether common causal factors underlie any such link requires longitudinal studies of older people. Few such studies have examined these issues, as highlighted in a recent review (Clouston et al. 2013). Results of prospective investigations of the relation between performance on different domains of fluid cognition and decline in walking speed have produced inconsistent findings (Soumare et al. 2009; Tabbarah et al. 2002; Watson et al. 2010), and while one study has demonstrated that slower usual walking speed is predictive of decline in attention and psychomotor speed (Inzitari et al. 2007), it is unclear whether slower walking speed is associated with change in other domains of fluid cognition. It is also uncertain whether changes in different domains of fluid cognition are associated with change in walking speed, although, in one cohort, less decline on a total cognitive score based in part on assessment of fluid cognitive abilities was associated with less decline in walking speed over a 7-year period (Tabbarah et al. 2002). In all these studies where a significant association remained after multivariate adjustment, effect sizes were small. Studies varied in the range of potential confounding variables included in the models, so the extent to which these factors account for the association remains unclear. Understanding the nature of the relationship between walking speed and different domains of fluid cognition in older people could help to inform the development of interventions to prevent or ameliorate mobility limitations and slow cognitive decline.

The English Longitudinal Study of Ageing (ELSA) is a large population-based sample of older men and women. We used these data to examine the dynamic relationship between fluid cognition, as measured by tests of executive function, verbal memory and processing speed, and usual walking speed over an average period of 6 years in people aged 60 to over 90 years.

## Methods

### Participants

The data for this study come from the ELSA. The sample for ELSA was based on people aged ≥50 years who had participated in the Health Survey for England in 1998, 1999 or 2001 (Steptoe et al. 2013). It was drawn by postcode sector, stratified by health authority and proportion of households in non-manual socioeconomic groups. In 2002–2003, 11,392 people participated in wave 1. Subsequent follow-up surveys have taken place approximately every 2 years. Ethical approval was obtained from the Multicentre Research and Ethics Committee. Participants gave written informed consent.

### Measures

#### Cognitive function

The ELSA data include scores on four tests of cognitive function taken at each follow-up occasion: verbal fluency, immediate and delayed verbal memory, and attention. Verbal (semantic) fluency was assessed by asking participants to name as many animals as they could think of in 1 min. Immediate and delayed verbal memory was assessed by presenting a list of 10 nouns aurally on a computer, one every 2 s. Participants were asked to recall as many words as possible immediately and again after a short delay during which they carried out the other cognitive tests. Attention and mental speed were assessed using a letter cancellation task. Participants were given a clipboard to which was attached a page of 780 random letters of the alphabet set out in a grid of 26 rows and 30 columns and were asked to cross out as many target letters (P and W) as possible in 1 min. An example was given at the top of the page to show participants how to cross out the letters. Participants were asked to work across and down the page as if they were reading and to perform the task as quickly and accurately as possible. Scores on these tests were used as measures of three domains of cognitive function: the score on the animal naming task was taken as a measure of executive function, the sum of the scores on the immediate and delayed recall tasks were taken as a measure of memory (these scores were highly correlated: *r* = 0.70 at wave 1), and the score for number of target letters correctly identified on the letter cancellation task was taken as a measure of processing speed/attention (termed processing speed hereafter). Each variable was centred on the variable’s mean at wave 1 and scaled into units of its standard deviation at wave 1.

#### Walking speed

Walking speed was assessed in participants aged 60 and over by measuring the time taken to walk a distance of 8 ft at usual pace. Before participants performed the test, the interviewer assessed whether they could do so safely. Use of walking aids such as a stick or Zimmer frame were permitted in the test; approximately 4 % of those who completed the test used these aids. Participants who had to rely on the support of another person or who were assessed as being in danger of falling were not asked to take the test. The timed walk was repeated and the mean of the two measurements was calculated.

#### Covariates

A priori, we identified potential confounding factors; that is factors previously shown to be associated with both cognitive function and walking speed in later life. These factors were socioeconomic status, educational attainment, height, adiposity, muscle strength, smoking, physical activity, chronic physical illness, blood pressure and depressive symptoms (Atkinson et al. 2005; Christensen et al. 1997; Deary et al. 2011; Demakakos et al. 2013; Dore et al. 2008; Elias et al. 1993; Guven and Lee 2013; Hardy et al. 2013; Kenny et al. 2013; Koster et al. 2005; Kramer et al. 2006; Nooyens et al. 2008; Rosano et al. 2011; Waldstein et al. 2001; Zaninotto et al. 2013).

Socioeconomic status was indexed by total household wealth, including savings and investments, value of any property or business assets, net of debt, excluding pension assets at wave 1. Household wealth has been identified as the most accurate indicator of long-term socioeconomic circumstances in ELSA (Banks et al. 2003). Educational attainment was defined by age at leaving full-time education. This was coded as a continuous linear scale 1 to 7 (see Table 1). Participants provided information about their smoking habits and responded to three questions about the frequency with which they did vigorous, moderate or mild exercise. We ranked the combinations of responses to these questions according to the amount and intensity of exercise involved to provide an estimate of usual physical activity. Symptoms of depression were assessed using the eight-item version of the Center for Epidemiologic Studies Depression Scale (CES-D) (Steffick and The HRS working group 2000). Participants were asked whether a doctor had ever told them that they had any of the following conditions: angina, heart attack, congestive heart failure, diabetes or high blood sugar, a stroke, chronic lung disease, arthritis or rheumatism, or cancer. For the purposes of these analyses, we created a single variable to indicate history of heart disease from the variables on angina, heart attack and congestive heart failure.

**Table 1:** Characteristics of the study participants at baseline (wave 1) (*n* = 2,654)

Characteristic	Mean (SD) or no. (%)
Age (year)	68.79 (6.33)
Female	1,476 (55.6)
Age finished full-time education	
Did not attend school	12 (0.5)
≤ 14	686 (26.4)
15	818 (31.5)
16	471 (18.1)
17	193 (7.4)
18	115 (4.4)
≥ 19	305 (11.7)
Household wealth (quintiles)	
1	379 (14.4)
2	487 (18.5)
3	550 (20.8)
4	577 (21.9)
5	645 (24.5)
Heart disease	342 (12.9)
Stroke	93 (3.5)
Diabetes	163 (6.1)
Chronic lung disease	156 (5.9)
Asthma	297 (11.2)
Arthritis	895 (33.7)
Osteoporosis	150 (5.7)
Cancer	173 (6.5)
Depressive symptom score	0.7 (0.54)
Smoking	
Never	1004 (37.8)
Ex-	1319 (49.7)
Current	331 (12.5)
Exercise	
Low	730 (27.5)
2	880 (33.2)
3	561 (21.1)
High	483 (18.2)
*Grip strength (kg)	30.2 (10.3)
*Height (cm)	164.7 (9.3)
*Waist–hip ratio	0.9 (0.08)
*BMI (kg/m^2^)	27.7 (4.49)
*Systolic blood pressure (mmHg)	138.1 (19.0)
*Diastolic blood pressure (mmHg)	74.3 (10.8)

*Data available at wave 2

The covariates were complete except for the following numbers of missing values: education 54, wealth 16, grip strength 303, height 338, waist–hip ratio 338, BMI 361, systolic and diastolic BP 542

At wave 2 in 2004-2005, participants who completed the main interview were invited to have a visit from a nurse that included anthropometric measurements and measurement of blood pressure and grip strength. Height and weight were measured with a portable stadiometer and electronic scales, respectively. Body mass index (BMI) was calculated as weight (in kilograms)/height (in metres)^2^. Blood pressure was measured three times on the right arm; mean systolic and diastolic blood pressure was calculated based on the second and third measurements only. Maximum handgrip strength was measured three times on each side using a dynamometer; the best of these measurements was used for analysis.

### Analytical sample

Of the 6,974 study members aged 60 to over 90 years who completed the interviews in person at wave 1, 3,708 (53 %) were interviewed at wave 4 in 2008-2009. We excluded cases who had reported by wave 1 that a doctor had told them that they had Alzheimer’s disease, Parkinson's disease, dementia, organic brain syndrome or serious memory impairment (*N* = 120). In the present analysis, we excluded cases with a monotonic pattern of missing values in any of the four time-varying outcomes. The sample then consisted of 2,654 people who had complete data at waves 1 and 4. No attempt was made to impute missing values. Instead, we carried out a sensitivity analysis in order to show the missingness was ignorable under maximum likelihood estimation. The sensitivity analysis derived bootstrapped confidence intervals around the parameters estimates of each model to explore the variation in parameters that could result if missing values were not missing but imputed to the limits of plausibility. In a two-stage procedure, we first estimated prediction intervals for each missing value using individual growth curves based on all available information from shrinkage estimates of each person's growth parameters. In the second stage, we created 50 data sets each with different imputed missing values by randomly sampling with uniform probability within the prediction intervals. The models were fitted to each of these data sets and the variation in the resulting parameters was assessed. The parameters estimated with missing values ignored were all within the bootstrapped confidence intervals, and the intervals indicated that the sign and significance of model parameters across the multiple imputed data sets was the same as was obtained with missing values ignored. Therefore, we judged the occurrence of monotone missingness to be ignorable.

#### Statistical methods

We used linear mixed effects models of the relationship between the rate of change of cognition and the rate of change of walking speed, and vice versa, in our four-wave longitudinal data. Models were fitted using the “lmer” function of R (version 3.0.0). We did not directly use time-varying predictors, but instead derived time-invariant predictors representing linear slope, as has been done previously (Atkinson et al. 2010). These were derived as the change per year in walking speed, and in each domain of fluid cognition, by subtracting the relevant scores at wave 4 from scores at wave 1 and dividing the difference by the time in years between the two testing occasions. To test the assumption of linearity, we fitted models of each of our outcome variables regressed on age and age squared for each person. We found the curvature term was significant (*p* < 0.05) in less than 4.5 % of cases, and therefore judged linear slopes to be acceptable. Initial models were adjusted for age, age-squared and sex; these entered the models as main effects since there were no significant interactions. In model 2, we further adjusted for educational attainment and household wealth. In model 3, we further adjusted for all remaining covariates: doctor diagnoses of heart disease, stroke, diabetes, hypertension, osteoporosis, arthritis or rheumatism, chronic lung disease, asthma or cancer, physical activity, smoking, depressive symptoms, BMI, waist-to-hip ratio, blood pressure and grip strength. All these covariates were treated as time-invariant.

## Results

Baseline demographic and health-related characteristics of the 2,654 men and women in the study sample are shown in Table 1.

Table 2 shows the Pearson correlations between the various measures of walking speed and cognition (at baseline, at follow-up and the change between those time points), together with mean (SD) values for each variable. At baseline and at follow-up, performance on each domain of cognition assessed—executive function, memory and processing speed—tended to be better in those with faster walking speed. On average, mean walking speed and mean scores for each domain of cognitive function assessed fell over the follow-up period. Change in walking speed was positively correlated with change in each domain of cognition assessed and the size of each of these correlation coefficients was almost identical (*r* ranged from 0.061 to 0.065), indicating that people with less decline in each domain of cognitive function since baseline tended to experience less slowing in walking speed. As expected, change in one domain of cognitive function was correlated with change in the other two domains, but the size of these correlation coefficients differed little from those observed with change in walking speed.

**Table 2:** Correlations between the measures of walking speed and domains of fluid cognition at baseline (W1) and follow-up (W4)

	1	2	3	4	5	6	7	8	9	10	11	12	Mean (SD)
1. Walking speed (m/s) W1	-												0.92 (0.27)
2. Walking speed (m/s) W4	0.542**	–											0.81 (0.28)
3. Executive function W1	0.242**	0.249**	–										19.57 (5.85)
4. Executive function W4	0.283**	0.347**	0.519**	–									19.03 (6.28)
5. Memory W1	0.237**	0.260**	0.366**	0.375**	–								9.53 (3.13)
6. Memory W4	0.242**	0.315**	0.339**	0.480**	0.520**	–							9.21 (3.34)
7. Processing speed W1	0.160**	0.173**	0.232**	0.224**	0.208**	0.208**	–						18.8 (5.50)
8. Processing speed W4	0.173**	0.248**	0.239**	0.308**	0.269**	0.310**	0.511**	–					17.57 (5.45)
9. Walking speed change	-0.453**	0.500**	0.016	0.076**	0.030	0.084**	0.019	0.081**	–				-0.06 (0.16)
10. Executive function change	0.061**	0.121**	-0.434**	0.542**	0.036	0.175**	0.011	0.101**	0.065**	–			-0.02 (0.17)
11. Memory change	0.027	0.084**	0.006	0.149**	-0.412**	0.561**	0.021	0.069**	0.062**	0.153**	–		-0.02 (0.17)
12. Processing speed change	0.014	0.074**	-0.002	0.085**	0.062**	0.101**	-0.494**	0.491**	0.061**	0.090**	0.045*	–	-0.04 (0.16)

**p < 0.01; *p < 0.05

Table 3 shows the results of mixed effects models of the relationship between a SD increment in scores on each domain of cognition at baseline and yearly change in walking speed between baseline and follow-up. In models adjusting for age and sex only, better performance in each domain of cognition at baseline was associated with less yearly decline in walking speed. The effect size was small: for a SD increment in executive function, memory or processing speed, the yearly decline in walking speed was smaller by 0.061, 0.047 or 0.036 of a SD. Further adjustment for education and household wealth attenuated effect sizes but they remained statistically significant. Further adjustment for the remaining covariates (anthropometric and health-related variables) weakened all associations still further such that the associations between baseline memory or processing speed and change in walking speed ceased to be significant. The relationship between baseline executive function and change in walking speed remained statistically significant.

**Table 3:** Relationship between scores on different domains of cognition at baseline and change in walking speed over the follow-up period

Predictor	Model^a^	Regression coefficient for change in walking speed		
		Coefficient	SE	*p* value
Cognition at baseline, per SD increment				
Executive function	1	0.061	0.010	<0.01
Memory	1	0.047	0.010	<0.01
Processing speed	1	0.036	0.010	<0.01
Executive function	2	0.032	0.010	<0.01
Memory	2	0.020	0.010	<0.01
Processing speed	2	0.025	0.011	<0.01
Executive function	3	0.025	0.011	0.023
Memory	3	0.018	0.012	0.115
Processing speed	3	0.017	0.011	0.123

^a^Models: 1—adjusted for age, age-squared and sex; 2—further adjusted for education, and household wealth at baseline; 3—further adjusted for self-reported doctor diagnoses of heart disease, stroke, diabetes, hypertension, osteoporosis, arthritis or rheumatism, chronic lung disease, asthma or cancer, physical activity, smoking, and depressive symptoms at baseline, plus BMI, waist-to-hip ratio, grip strength and blood pressure at wave 2

Table 4 shows results of models of baseline walking speed in relation to yearly change in performance on each domain of cognition by the end of follow-up. In models adjusting for age and sex only, faster walking speed was associated with significantly less yearly decline in each domain of cognition: a SD increment in walking speed was associated with 0.076, 0.065 and 0.050 SD less yearly decline in executive function, memory and processing speed, respectively. Further adjustment first for education and household wealth and then in addition for anthropometric and health-related variables attenuated effects but all associations remained statistically significant.

**Table 4:** Relationship between walking speed at baseline and change in different domains of cognition over the follow-up period

Predictor	Model^a^	Regression coefficient for change in cognition			
		Cognitive domain	Coefficient	SE	*p* value
Walking speed at baseline, per SD increment	1	Executive function	0.076	0.010	<0.01
	1	Memory	0.065	0.011	<0.01
	1	Processing speed	0.050	0.009	<0.01
	2	Executive function	0.045	0.011	<0.01
	2	Memory	0.041	0.011	<0.01
	2	Processing speed	0.037	0.010	<0.01
	3	Executive function	0.036	0.013	<0.01
	3	Memory	0.031	0.013	0.015
	3	Processing speed	0.025	0.012	0.038

^a^Models: 1—adjusted for age, age-squared and sex; 2—further adjusted for education, and household wealth at baseline; 3—further adjusted for self-reported doctor diagnoses of heart disease, stroke, diabetes, hypertension, osteoporosis, arthritis or rheumatism, chronic lung disease, asthma or cancer, physical activity, smoking, and depressive symptoms at baseline, plus BMI, waist-to-hip ratio, grip strength and blood pressure at wave 2

Table 5 shows results of equivalent models of the relationship between yearly change in each domain of cognition and yearly change in walking speed over the follow-up period. Less yearly decline in each domain of cognition was associated with less yearly decline in walking speed. In models adjusting for age and sex, a SD less yearly decline in executive function, memory or processing speed was associated with 0.058, 0.046 and 0.047 SD less yearly decline in walking speed, respectively. Further adjustment for education and household wealth had small attenuating effects and all associations remained statistically significant. After additional adjustment for the anthropometric and health-related variables, the association between yearly decline in memory and yearly decline in walking speed was no longer significant, but the associations between yearly decline in executive function or processing speed and yearly decline in walking speed remained significant.

**Table 5:** Relationship between change in different domains of cognition and change in walking speed over the follow-up period

Predictor	Model^a^	Regression coefficient for change in walking speed		
		Coefficient	SE	*p* value
Change in cognition, per SD increment				
Executive function	1	0.058	0.011	<0.01
Memory	1	0.046	0.011	<0.01
Processing speed	1	0.047	0.011	<0.01
Executive function	2	0.036	0.011	<0.01
Memory	2	0.023	0.011	0.040
Processing speed	2	0.029	0.011	0.011
Executive function	3	0.036	0.012	<0.01
Memory	3	0.011	0.012	0.378
Processing speed	3	0.025	0.012	0.041

^a^Models: 1—adjusted for age, age-squared and sex; 2—further adjusted for education, and household wealth at baseline; 3—further adjusted for self-reported doctor diagnoses of heart disease, stroke, diabetes, hypertension, osteoporosis, arthritis or rheumatism, chronic lung disease, asthma or cancer, physical activity, smoking, and depressive symptoms at baseline, plus BMI, waist-to-hip ratio, grip strength and blood pressure at wave 2

## Discussion

In this prospective study of 2,654 men and women aged 60 to over 90 years, there was a bidirectional relationship between walking speed and fluid cognition. After adjusting for age and sex, people with better baseline performance on tests of executive function, memory and processing speed experienced less yearly decline in walking speed over the average 6-year follow-up period; similarly, people who walked faster at baseline experienced less yearly decline on each cognitive domain. Extent of yearly change in each cognitive domain over the follow-up period was associated with extent of yearly change in walking speed. Effect sizes were small, ranging from 0.04 to 0.06 of a SD. After further adjustment for education, household wealth, anthropometric and health-related factors, associations were weakened but most remained statistically significant.

### Comparison with previous studies

Most of the few previous studies into the longitudinal associations between fluid cognitive abilities and walking speed in older people have focussed on cognition as a predictor of decline in walking speed (Clouston et al. 2013). They found that greater decline in walking speed was associated with poorer baseline performance on some, if not all, domains of fluid cognition (Soumare et al. 2009; Watson et al. 2010) or with decline in cognition, based on a combined score on tests of several domains (Tabbarah et al. 2002). Greater decline in walking speed has also been linked with poorer baseline performance or greater change in score on the Modified Mini-Mental State Examination (Atkinson et al. 2010). In our study, as in those described above (Soumare et al. 2009; Watson et al. 2010), the difference in effect size between those domains of fluid cognition that were significantly associated with decline in walking speed and those that were not was very small. Evidence from a small pilot randomized controlled trial of cognitive training, focussed primarily on executive function and attention but also visuospatial skills and processing speed, suggests that improving performance in these domains of cognition may lead to increases in walking speed in older people (Verghese et al. 2010). Whether the beneficial effect seen during this trial was due to improvements in a specific cognitive domain or in the multiple domains targeted remains unclear.

There have been few previous investigations into whether walking speed in linked with change in particular domains of fluid cognition (Clouston et al. 2013), and evidence that it is predictive of change in score on the Mini Mental State Examination have been inconsistent (Alfaro-Acha et al. 2007; Atkinson et al. 2010; Auyeung et al. 2011; Deshpande et al. 2009). In the Health, Aging and Body Composition Study, participants with slower walking speed experienced a significant decline in attention and psychomotor speed, as measured by performance on the Digit Symbol Substitution test (Inzitari et al. 2007). Our findings suggest that slower walking speed may also be a risk factor for declines in other domains of fluid cognition. However, it is worth noting that walking speed is just one of many measurable facets of gait. There is some evidence that facets of gait may differ in the strength with which they are linked to specific domains of cognition. In a longitudinal study which used factor analysis of eight different gait measures in non-demented older people, the resulting ‘pace’ and ‘rhythm’ and ‘variability’ factors were not equally predictive of cognitive decline in different domains; worse performance on the ‘pace’ factor—on which walking speed and stride length loaded highly—was associated specifically with decline in executive function, while worse performance on ‘rhythm’—primarily a reflection of cadence, swing time and stance time—was associated specifically with decline in memory (Verghese et al. 2007).

### Potential mechanisms

The associations we observed in this study were attenuated after adjustment for covariates, including education, household wealth, anthropometric and health-related factors, but most associations persisted. It may be that brain small vessel disease is a common aetiology underlying these bi-directional associations. There is considerable evidence of the role that white matter damage and brain atrophy play in age-related cognitive decline (Debette and Markus 2010; Jokinen et al. 2012; Schmidt et al. 2007). Cross-sectional observations in the Cardiovascular Health Study have shown that slower walking speed is linked with a higher prevalence of white matter disease and subclinical strokes in older people who were free from dementia (Rosano et al. 2006), and a recent longitudinal investigation from the Tasmanian Study of Cognition and Gait demonstrated that structural changes in the brain, such as white matter and hippocampal atrophy and increasing white matter lesions, were associated with decline in walking speed in older people (Callisaya et al. 2013). Another possibility may be that some common biological process of cellular senescence underlies the bidirectional association between cognition and walking speed (Campisi and d'Adda di Fagagna 2007). Cellular senescence is an established cellular stress response to prevent proliferation of cells exposed to potentially oncogenic stimuli. Senescent cells occur with increasing frequency in older tissues and it has been hypothesized that the secretion of numerous pro-inflammatory cytokines, growth factors and proteases that accompanies cellular senescence may be implicated in a variety of age-related pathologies, including decline in cognitive and motor function (Campisi et al. 2011).

### Strengths and limitations

The strengths of our study include the large sample size, the fact that it is representative of the community-dwelling English population aged 60 and over (Taylor et al. 2003), and the availability of information on a range of potential confounding. There are also some weaknesses. Firstly, a small proportion of people (around 8 % at wave 1) did not do the walking speed test because they were unable to walk alone or had health restrictions. This may mean that our results under-estimate the strength of association between cognition and walking speed. Secondly, we had no data on other facets of gait and walking speed was assessed under only one condition—usual pace—so we were unable to examine whether the associations we found with domains of cognition were consistent across different facets of gait or whether they varied with the condition under which the timed walk was undertaken. In one study, fastest walking speed was more strongly predictive of cognitive decline on the MMSE than usual walking speed (Deshpande et al. 2009). Thirdly, the tests of cognitive function used in ELSA were brief and may as a consequence be less reliable; this may have resulted in under-estimation of effect sizes. Fourthly, we treated all covariates as time-invariant; thus, we take account of the potential role that initial levels of these covariates might play in the longitudinal bidirectional associations between cognitive ability and walking speed, but we are not able to tell whether change in some of these covariates over the short follow-up period might play a part in these associations. Finally, in common with other longitudinal studies, especially in people of the age studied here, there was attrition over time and missing data on some covariates. In order to minimize the potential bias due to missing data, we used full information maximum likelihood estimation. Comparison of the proportions of the response levels within each covariate remained reasonably constant across waves. This suggests that it is reasonable to assume that data are missing at random and that no systematic bias has been introduced.

In this prospective study of men and women aged 60 to over 90 years, we found bidirectional associations between better performance on tests of different domains of fluid cognition and faster walking speed that were for the most part only partially explained by our covariates. There was some evidence that walking speed and the cognitive domains of executive function and processing speed may change in parallel with increasing age. As with previous investigations into this association based solely on older people, we were not able to ascertain whether change in walking speed precedes change in cognition or vice versa. Longitudinal data on cognition and walking speed from much earlier in life would give us a clearer understanding of the origins of this relationship and how it changes with age. It might also inform the development and timing of interventions.
